# Enhanced functionalities of biomaterials through metal ion surface modification

**DOI:** 10.3389/fbioe.2025.1522442

**Published:** 2025-04-14

**Authors:** Yujie Tao, Wayne Nishio Ayre, Liming Jiang, Siyu Chen, Yuqi Dong, Lin Wu, Yilai Jiao, Xiaohan Liu

**Affiliations:** ^1^ School and Hospital of Stomatology, China Medical University, Liaoning Provincial Key Laboratory of Oral Diseases, Shenyang, China; ^2^ School of Dentistry, Cardiff University, Cardiff, United Kingdom; ^3^ Chinese Academy of Sciences Shenyang Branch, Shenyang, China

**Keywords:** bone defect repair, metal ions, osteogenesis, antibacterial activity, biomaterials

## Abstract

The development of new artificial biomaterials for bone defect repair is an ongoing area of clinical research. Metal ions such as zinc, copper, magnesium, calcium, strontium, silver, and cerium play various roles in bone tissue regeneration in the human body and possess a range of biochemical functions. Studies have demonstrated that appropriate concentrations of these metal ions can promote osteogenesis and angiogenesis, inhibit osteoclast activity, and deter bacterial infections. Researchers have incorporated metal ions into biomaterials using various methods to create artificial bone materials with enhanced osteogenic and antibacterial capabilities. In addition to the osteogenic properties of all the aforementioned metal ions, Zn, Sr, and Ce can indirectly promote osteogenesis by inhibiting osteoclast activity. Cu, Mg, and Sr significantly enhance angiogenesis, while the antibacterial properties of Zn, Cu, Ag, and Ce can reduce the likelihood of infection and inflammation caused by implanted materials. This paper reviews the mechanisms through which metal ions promote bone tissue growth and improve the antibacterial activity of biomaterials. It also summarizes common loading methods on the surface of biomaterials with different metals and highlights the potential clinical applications of these new artificial bone materials.

## 1 Introduction

Bone is an organ with complex layers whose main functions are to protect vital organs and provide the body with mechanical stability (Florencio-Silva et al., 2015). Bone defects caused by congenital malformations, trauma, infection, and surgery can impose significant physical and psychological burdens on patients and are associated with high costs, resulting in economic pressures on medical institutions and health services (Wang S. et al., 2021). Currently, the main methods for repairing bone defects include autografts, allografts, and the use of artificial materials. Among these options, autografts are considered the “gold standard” for treating large bone defects due to their strong ability to promote bone regeneration and their low risk of immune rejection (Sakkas et al., 2017). Allografts have the advantage of avoiding damage to the donor site and are not limited by the size of the grafted bone (Li S. et al., 2022). However, both methods have shortcomings: autografts have a limited supply of available bone tissue, and allografts may lead to disease transmission or immune rejection. Therefore, there is a need for effective, synthetic, non-immunogenic, and readily available implant materials capable of regenerating bone tissue for the clinical treatment of bone defects.

Within the field of bone biomaterials, metallic elements and their compounds have been widely investigated for the repair of bone defects. Studies have shown that metal ions possess excellent stability and biosafety, and that appropriate concentrations of metal ions can promote osteogenesis and angiogenesis, inhibit the activity of osteoclasts and bacteria, participate in various biochemical functions in the human body, and play crucial roles in different aspects of bone tissue regeneration (Glenske et al., 2018; Bosch-Rué et al., 2021; Li S. et al., 2022). Regeneration of bone tissue is a process in which cells from different lineages interact with others to promote tissue healing. Osteoblasts and osteoclasts are the two main cells in the process of bone remodeling, which regulate the growth and resorption of bone tissue, and the interaction of these 2 cells plays vital importance in the homeostasis of bone remodeling (Zhang et al., 2022b). Studies have shown that osteoblasts can regulate the differentiation and osteoclast apoptosis through direct cell–cell communication, the binding between membrane-bound ligands and receptors, or the release of soluble factors (Matsuo and Irie, 2008). In turn, osteoclasts can influence the activity of osteoblasts and promote osteogenic differentiation by secreting coupling factors, known as clastokines (Daponte et al., 2024). Angiogenesis also plays an important role in the formation of new bone by transporting nutrients and growth factors, as well as releasing paracrine signals that regulate the growth, differentiation and regeneration of different cell types, including osteoblasts (Diomede et al., 2020). Studies have shown that vascular endothelial growth factor (VEGF), a prototypic angiogenic factor, is highly expressed in periosteum and chondrocytes, and can stimulate the expression of osteogenic factors, enhancing bone mass accumulation and bone strength (Towler, 2003; Grellier et al., 2009). Additionally, infections that occur during bone regeneration pose a significant challenge, bacteria will form biofilms on the surface of the implant that are difficult to be destroyed by antimicrobial agents, such infections will impair bone integration and even lead to the failure of implantation, therefore it is necessary to endow antibacterial properties to materials to prevent the initial adhesion and proliferation of bacteria (Hu et al., 2012a).

Researchers have incorporated metal ions onto the surfaces of implant materials through various surface modification techniques to develop artificial bone materials with enhanced osteogenic and antibacterial capabilities. This article summarizes the possible mechanisms of action and common preparation methods of currently used metallic ions such as zinc (Zn), copper (Cu), magnesium (Mg), calcium (Ca), strontium (Sr), silver (Ag), and cerium (Ce) in improving the osteogenic and antibacterial activity of implant surfaces.

## 2 Zinc

Zn is commonly found in a variety of human tissues. Zinc content in adults is usually 1.4–2.3 g, and about 85% of zinc is found in muscle and bone (Chen B. et al., 2024). As an essential trace element, Zn plays a crucial role in the normal functioning of the human immune system, cell division, and the formation, development, and mineralization of bone (Glenske et al., 2018; Wang S. et al., 2021). It is estimated that zinc binds to about 3,000 proteins in the body, accounting for about 10% of the human proteome, and more than 3% of genes in the human body code for proteins containing zinc finger domains (Chen K. et al., 2024).

### 2.1 Osteogenic effects of zinc

Zn plays a crucial role in bone development and growth. Numerous studies have shown that zinc ions (Zn^2+^) can promote the osteogenesis of bone marrow mesenchymal stem cells (BMMSCs). As an essential cofactor for key enzymes related to bone metabolism, Zn^2+^ participates in the energy metabolism of bone cells, including upregulating the expression of alkaline phosphatase (ALP, an osteogenic factors, widely distributed in bone tissue), collagenase, and carbonic anhydrase (an enzyme that catalyzes the reversible reactions of carbon dioxide and water) (Maret, 2013; Yu et al., 2020). Zn^2+^ also promotes the expression of osteoblast markers such as osteocalcin (OCN), osteopontin (OPN), and runt-related transcription factor 2 (Runx2), as well as collagen type I (Col-1) by BMMSCs, thereby accelerating the proliferation and mineralization of osteoblasts (Li H. et al., 2022). Additionally, Runx2 is the earliest osteogenic marker, which can bind to OCN promoters and subsequently induces osteogenic marker genes ALP and Col-1 in early differentiation, and OCN in late differentiation (Yu et al., 2017).

The main signaling pathways that Zn regulates osteogenesis are the TGF-β/Smad and the mitogen-activated protein kinase (MAPK) pathways, with TGF-β being a key mediator for osteoblast development and MAPK (regulating the responses of monocytes to exogenous stress) being a bridge between the immune system and bone metabolism (Song et al., 2020; Xia et al., 2020; Li S. et al., 2022). TGF-β influences bone formation by inducing the phosphorylation of a set of intracellular transcription factors (known as Smads, among which Smad2/3 serves as a substrate for TGF-β receptors) through activating its receptors, and the activated Smad complex can then enter the nucleus to trigger the transcription of target genes (Heldin et al., 1997). Yu et al. prepared Zn-modified calcium silicate coatings on the surface of Ti-6Al-4V, and found that the Zn-containing coating significantly upregulated the expression of TGF-β1, Smad2 and Smad3 genes, therefore the activation of TGF-β/Smad signaling pathway promoted osteogenic differentiation and mineralization of bone marrow-derived pericytes (Yu et al., 2017). Both extracellular signal-regulated protein kinases 1 and 2 (ERK 1/2) and p38 are MAPK signaling pathway related proteins. Zn has been shown to promote osteogenic differentiation of BMMSCs by activating the p38 and ERK1/2 signaling pathways (Song et al., 2020). Additionally, Zn also has an osteogenic effect through the activation of the cAMP-PKA-CREB signaling pathway. It can promote the expression of intracellular cyclic adenosine monophosphate (cAMP), further enhance the activity of protein kinase A (PKA) and promote the translocation of phosphorylated cAMP-response element binding protein (CREB) into the nucleus, which ultimately enhances the expression of Runx2 and promotes osteoblast differentiation (Park et al., 2018).

However, the effects of Zn^2+^ on osteogenic differentiation can vary depending on its concentration. Yu et al. demonstrated that an appropriate concentration of Zn^2+^ in the bone microenvironment (2–5 μg/mL) effectively enhances the initial adhesion and proliferation of BMMSCs and promotes their differentiation toward osteoblasts by activating the MAPK/ERK signaling pathway without cytotoxicity. Conversely, a higher concentration of Zn^2+^ (15 μg/mL) was found to inhibit the adhesion, proliferation, and osteogenic differentiation of BMMSCs due to the disruption of the dynamic balance of intracellular and extracellular Zn^2+^ (Yu et al., 2020).

### 2.2 Inhibitory effects of zinc on osteoclasts

**FIGURE 1:**
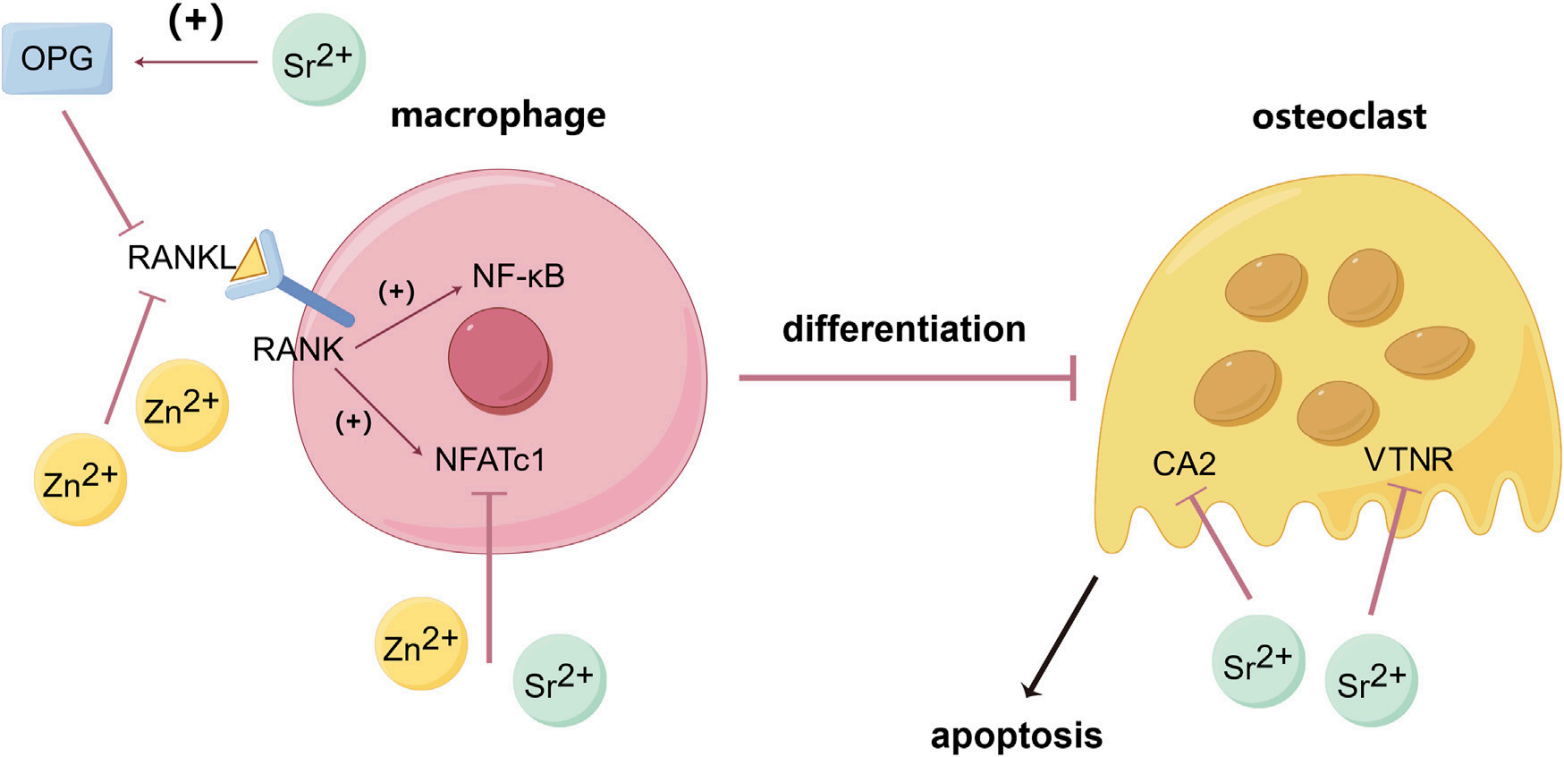
Inhibitory effects of Zn and Sr on osteoclastogenesis and osteoclast activity. (By Figdraw). Zn^2+^ can inhibit the activity of calcineurin lead to the reduction of NFATc1, and by suppressing the expression of RANKL in osteoblasts then decrease the binding between RANKL and RANK on the membrane of macrophage, thereby reducing the expression of NF-κB and NFATc1 and inhibiting osteoclastogenesis. Sr^2+^ can upregulate the expression of OPG thus blocking the interaction between RANKL and RANK, and by down-regulating the expression of carbonic anhydrase II (CA2, a key enzyme in bone resorption) and vitronectin receptor (VTNR, involved in cell capsule fold formation), thereby reducing osteoclast differentiation and activity. Bose et al. (2013), O’Connor et al. (2020), Lee et al. (2022), Li S. et al. (2022).

The homeostasis of bone in the human body requires a dynamic balance between osteoblasts and osteoclasts, which regulate the growth and resorption of bone tissue (Chen et al., 2018). Receptor activator of nuclear factor kappa-B ligand (RANKL) is a crucial factor in the differentiation of osteoclasts. Nuclear factor kappa B (NF-κB), the MAPK signaling pathway, and the nuclear factor of activated T cells (NFATc) play significant roles in promoting osteoclast maturation. When RANKL binds to RANK, it can activate NF-κB, MAPK signaling pathway, and NFATc to promote the differentiation into osteoclasts from macrophages (He S. et al., 2020; Hong et al., 2020; O’Connor et al., 2020). Studies have shown that Zn^2+^ can inhibit the activity of calcineurin (a Ca^2+^ and calmodulin-dependent serine/threonine protein phosphatase), reduce the expression level of NFATc1, and inhibit osteoclastogenesis. Zn^2+^ can also suppress the expression of RANKL in osteoblasts, thereby inhibiting osteoclastogenesis (O’Connor et al., 2020; Li S. et al., 2022) (Figure 1).

### 2.3 Antibacterial effects of zinc

**FIGURE 2:**
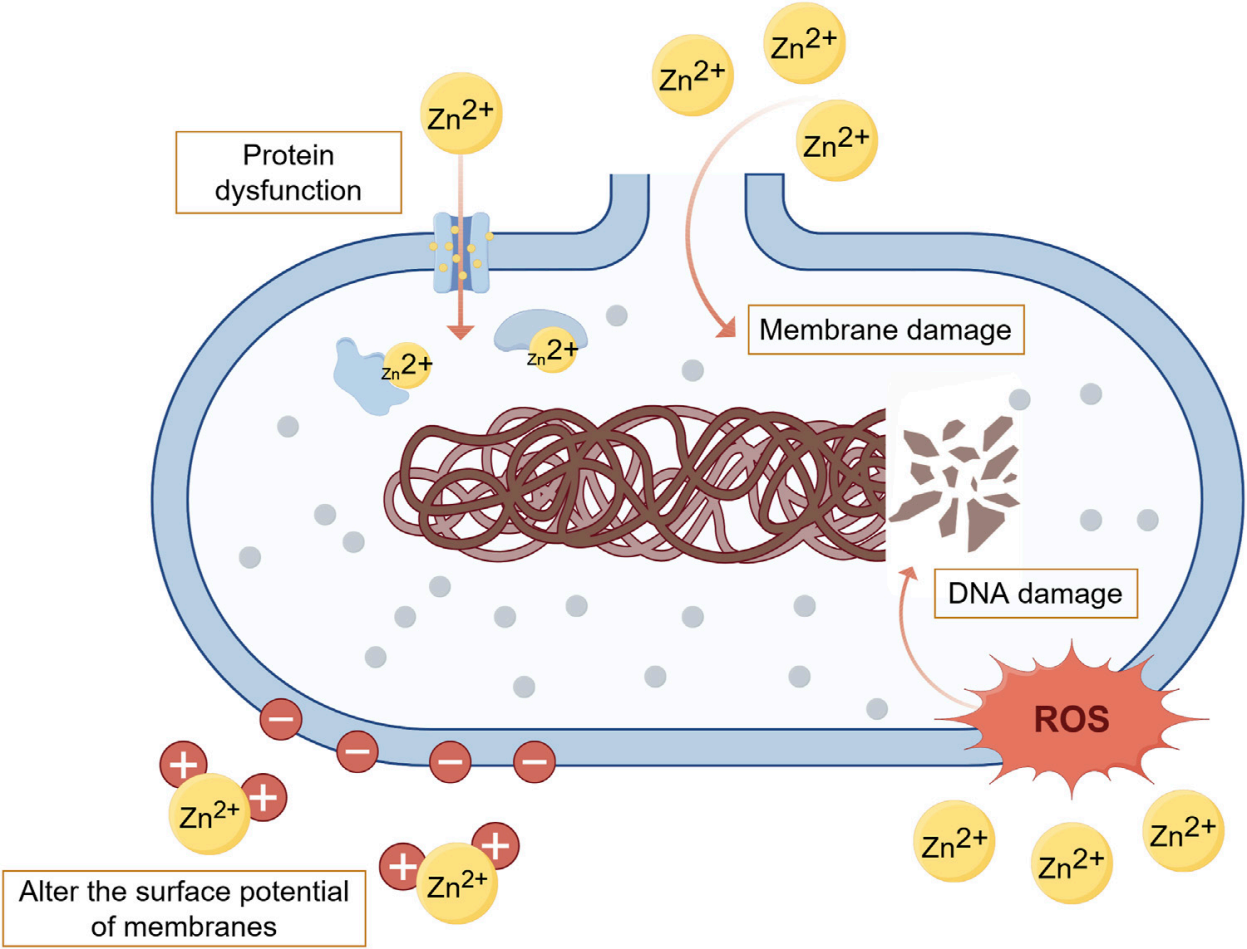
Main antibacterial mechanisms of Zn. (By Figdraw). Zn exerts its antibacterial effects mainly through three pathways: altering bacterial cell membrane permeability, interfering with bacterial metabolic processes, and inducing the production of ROS. Li et al. (2020), Jia et al. (2022), Shokri et al. (2022).

In addition to promoting osteogenesis and inhibiting osteolysis, Zn also demonstrates significant antibacterial properties through three pathways (Figure 2). Firstly, Zn^2+^ can alter the surface potential of bacterial cell membranes and increase membrane permeability, leading to the rupture and death of bacterial cells (Li et al., 2020). Secondly, Zn^2+^ enters bacterial cells through transmembrane proteins, affecting the synthesis of key proteins and enzymatic reactions involved in cellular metabolic processes, ultimately resulting in bacterial death (Li et al., 2020; Jia et al., 2022). Thirdly, Zn^2+^ can induce the production of reactive oxygen species (ROS) through active redox cycling on ZnO nanoparticle surfaces. Excessive ROS can cause oxidative stress and damage to lipids, DNA and proteins, leading to bacterial cell death (Jia et al., 2022; Shokri et al., 2022). Hu et al. used *E. coli* (*Escherichia coli*) as a test strain to evaluate the antimicrobial performance of Zn^2+^-containing coating materials. The results showed that the number of *E. coli* colonies on the surface of the Zn^2+^-containing materials was significantly reduced, with the number of colonies inversely proportional to the Zn^2+^ content in the coating, confirming that Zn^2+^-containing coatings possess excellent antibacterial properties (Hu et al., 2022). Hu et al. prepared Zn-incorporated TiO_2_ coatings on Ti using Plasma electrolytic oxidation (PEO). The results demonstrated that the coatings significantly inhibited the survival of *E. coli* and *S. aureus*, with bacterial survival inversely proportional to the concentration of Zn^2+^ (Hu et al., 2012b). Additionally, to avoid cytotoxicity from antibacterial concentrations of Zn^2+^, it is important to strictly control the concentration and release of Zn^2+^. Jin et al. prepared different concentrations (0.12–0.26 μg/mL) of Zn^2+^ coating on the surface of Ti to assess their impact on osteogenesis and antibacterial activity. The results demonstrated that four groups of Zn^2+^-containing samples (the concentrations were 0.12, 0.18, 0.23 and 0.26 μg/mL respectively) all had significant inhibitory effect on the growth of *E*. *coli* and *Staphylococcus aureus* (*S*. *aureus*), and the above concentration of Zn^2+^ can also promote the proliferation of MC3T3-E1 cells, enhance cell adhesion, increase ALP activity, and stimulate collagen secretion and extracellular matrix (ECM) mineralization of MSCs without inducing cytotoxicity (Jin et al., 2014).

### 2.4 Common preparation methods of biomaterials loaded with zinc

Zn has been widely used as a coating material for artificial bone due to its osteo-promoting and antibacterial properties. Zn can be incorporated into artificial bone materials through various methods, including laser printing, melt deposition, plasma surface modification and mechanochemical activation (Xu et al., 2022). Through the addition of Zn onto polyetheretherketone (PEEK) using a plasma-induced surface modification method, the Zn^2+^ in the coating showed a sudden release within the first 3 days. Zhang et al. found that the modified samples exhibited enhanced adhesion, proliferation, and differentiation of MC3T3-E1 cells, while inhibiting the activity of *S*. *aureus*, a common pathogen in joint replacement infections (Zhang Y. et al., 2021). Through the addition of zein/Zn-Mn mesoporous bioactive glass nanoparticle (MBGN) on 316L stainless steel (SS) by electrophoretic deposition (EPD), a strong hydrogen bond was formed between the composite coating, which makes it have excellent adhesion strength. Batool et al. also observed that the combination of Zn and Mn in the coating showed a continuous linear release trend and significant antibacterial and osteogenic properties (Batool et al., 2022).

Zn can also be modified on the surface of Ti implants through electrochemical deposition, magnetron sputtering, micro-arc oxidation (MAO), and hydrothermal (HT) methods to create biomaterials with excellent mechanical properties, biocompatibility and osteogenic and antibacterial activities (Wang Z. et al., 2021). Zhao et al. used anodic oxidation electrochemical insertion to construct Zn-doped titanium dioxide (TiO_2_) nanotube arrays on the surface of Ti implants. The analysis by electron probe microanalyzer (EPMA) showed that Zn had been successfully inserted into the TiO_2_ nanotube and showed a stable and linear release trend within 12 days. The incorporation of Zn^2+^ improved the bioactivity of Ti implants and enhanced the attachment, proliferation, and osteogenic differentiation of mesenchymal stem cells (MSCs) (Zhao H. et al., 2022). EL-Wassefy et al. prepared a Zn-doped HA coating on the surface of Ti using an electrochemical deposition method, the coating is rosette-shaped under the microscope and is stably attached to the substrate surface, which significantly increased the surface area, surface roughness and initial stability of the Ti implant (El-Wassefy et al., 2017). Additionally, Zn can be combined with other metals, such as Cu and Ag, to modify the surfaces of polymers or Ti, further enhancing osteogenic and antibacterial activities (Qu et al., 2021; Wang S. et al., 2021).

## 3 Copper

Cu is a crucial trace element in the human body, known for its excellent biosafety, and it has been widely utilized in biomedical materials. Cu assists in protein transfer and plays a vital catalytic role in the formation of heme, a molecular component of hemoglobin, which binds oxygen in the bloodstream, and a deficiency of copper can lead to anemia, bone abnormalities and arterial issues (Bertinato and L’Abbé, 2004). Additionally, Cu is essential in many enzymes and plays a key role as a cofactor in a variety of biological processes including superoxide dismutase, amine oxidase and ceruloplasmin, it can also be used as a cofactor to intermingle with the amino acid side chain of proteins, thereby regulating complex cellular activities (Chen et al., 2023).

### 3.1 Osteogenic and angiogenic effects of copper

Studies have shown that Cu promotes osteogenesis by enhancing angiogenesis and collagen deposition in the defect area (Gérard et al., 2010). Lack of Cu may reduce the cross-linking of collagen, which leads to the increased fragility of bones (Shi et al., 2016). This effect is based on encouraging cells to produce higher levels of angiogenic factors and stimulating the expression of angiogenesis-related genes. Copper ions (Cu^2+^) can reduce oxygen tension around histiocytes to mimic a hypoxic environment or stabilize the expression of hypoxia-inducible factor-1α (HIF-1α). HIF is a key regulator of hypoxic response, with transcriptional targets include genes in angiogenesis, vasomotor function, and apoptosis/proliferation response (Kaelin, 2005). Under local hypoxia, histiocytes produce higher levels of angiogenic factors such as VEGF, HIF-1 and erythropoietin (EPO), Cu^2+^ also stimulates the expression of angiogenesis-related genes and increases VEGF-A protein secretion in the meantime, both leading to the formation of more blood vessels and blood cells to counteract hypoxia and thereby indirectly promoting osteogenesis (Lin et al., 2020; Li S. et al., 2022; Noori et al., 2022). Cu^2+^ can also regulate the transcription of angiopoietin-1 (ANG-1, mainly expressed by perivascular cells involved in vascular stabilization and neovascularization) and can promote the transport of ANG from the extracellular to the intracellular compartment and its enrichment in cytoplasm and around nucleus (Giacomelli et al., 2015; Jacobs et al., 2020). Studies also found that lower concentration of Cu^2+^ can stimulate the activity of matrix metalloproteinases (MMPs, involved in the wound healing process, can regulating the activity of growth factors such as VEGF), while higher concentrations of Cu^2+^ can increase the expression of MMPs in fibroblasts (Jacobs et al., 2020). Additionally, Cu^2+^ can regulate the conversion of macrophages from the M1 (pro-inflammatory) to the M2 (anti-inflammatory) phenotype by activating the STAT6 pathway (signal transducer and activator of transcription 6, a key transcription factor for M2 polarization) and inducing the expression of M2-related genes (such as IL-10, TGF-β) or activating the PI3K/Akt signaling pathway and promoting metabolic reprogramming of fatty acid oxidation and glycolysis (a typical metabolic feature of M2 macrophages), with M2 macrophages expressing higher levels of platelet-derived growth factor (PDGF) and VEGF, thus promoting angiogenesis (Han et al., 2022). On the other hand, Cu can also stimulate the osteogenic differentiation and ECM mineralization of osteoblast precursor cells (Westhauser et al., 2021). Liang et al. prepared Cu coatings on the surface of pure Mg, which significantly improved the ALP activity, upregulated the expression of OCN and Col-1 and promoted the formation of mineralized nodules in MC3T3-E1 cells, thereby enhancing the osteogenic differentiation (Liang et al., 2021).

### 3.2 Antibacterial effects of copper

Cu^2+^ is frequently chosen as the preferred metal ion for biomaterials surface modification due to its broad-spectrum antibacterial properties (Duan et al., 2022). The antibacterial mechanism of Cu^2+^ may include altering the microenvironment of bacteria, inhibiting the respiration of bacterial cells, and destroying bacterial cell membranes. The bactericidal effect of Cu^2+^ mainly performs as follows. Cu^2+^ can perforate cell membranes, leading to bacterial rupture; disrupt proteins related to membrane transport, causing ion concentration changes inside and outside the cell, thus killing bacteria (Zhao et al., 2019). Additionally, similar to Zn^2+^, Cu^2+^ can produce ROS, inhibit 16SrRNA replication, and cause bacterial DNA damage, resulting in bacterial death (Fowler et al., 2019). Cu^2+^ also has bacteriostatic effect. It can bind to bacterial DNA, inhibiting DNA polymerase synthesis, thereby hindering bacterial proliferation (Li M. et al., 2016). Yuzer et al. tested the antibacterial properties of Cu-containing coatings using *S. aureus* and *E. coli*. The results showed that, compared to the control group, Cu-containing coatings exhibited superior antibacterial effects, with the antimicrobial efficacy proportional to the Cu^2+^ concentration (samples with 5% Cu had about twice the antimicrobial effect of undoped samples) (Yuzer et al., 2022). However, since high concentration of Cu^2+^ can be cytotoxic, Ines et al. compared the effects of different concentrations of Cu-coated Ti-6Al-4Von MSCs and bacteria under the same culture conditions. The results showed that when the concentration of Cu^2+^ reached 0.5mM, it would be cytotoxic to MSCs, but 0.3 mM Cu^2+^ could significantly inhibit the growth and proliferation of bacteria, indicating that an appropriate concentration of Cu^2+^ could achieve good antibacterial effect without causing cytotoxicity (Burghardt et al., 2015).

### 3.3 Common preparation methods of biomaterials loaded with copper

In biomaterials, Cu can be used alone or in combination with other elements to achieve excellent osteogenic, angiogenic, and antibacterial properties. Common methods for preparing Cu-loaded biomaterials include MAO, *in situ* chemical reduction, anodic oxidation, and HT treatment. Through the addition of Cu to TiO_2_ microporous coatings on Ti implants using MAO, Kang et al. found that the coating showed a crater-like porous structure under microscope and the XPS results show that the Cu and O bond combination in the form of CuO. The Cu^2+^ on the surface of Ti implant showed a tendency of rapid release at the beginning, then maintained a low level of steady release (Kang et al., 2022). Through the addition of Cu^2+^ and Mg^2+^ to TiO_2_ nanotube coating Ti by anodic oxidation and HT, Wang et al. found that the coating appeared as a nanoscale network structure, close to the shape of natural human bone, which is conducive to cell proliferation. And the release kinetics curves of Mg^2+^ and Cu^2+^ follow the below rules: burst release, sustained release and near-linear release (Wang B. et al., 2021). Yang et al. used *in situ* chemical reduction to prepare graphene oxide/copper (GO/Cu) nanocomposite coatings on chitosan/hyaluronic acid scaffolds. The electrostatic interaction between Cu^2+^ and charged oxygen-containing groups provided nucleation sites for the anchor and growth of Cu nanoparticles (CuNPs), and the Cu in the coating was released in both Cu^2+^ and CuNPs forms which exhibited synergistic antibacterial effects and good osteogenic property (Yang et al., 2022).

## 4 Calcium

Ca, the most prevalent mineral in the human body, is the primary inorganic component of bones and teeth and is involved in various physiological activities such as bone growth, muscle contraction and nerve transmission (Clapham, 2007). Plasma Ca homeostasis plays a crucial role in maintaining human life activities, such as maintaining of the bones, regulating hormone secretion, transmission of nerve impulses and blood vessel activity, and homeostasis of Ca is mainly maintained by parathyroid hormone (PTH) and calcitonin (Li et al., 2018b). When serum Ca levels decrease, PTH promotes Ca release from bone and stimulates calcium reabsorption by renal tubules; reversely when serum Ca levels are elevated, calcitonin inhibits the release of Ca from bones and reduces the reabsorption of Ca by renal tubules (Baird, 2011; Li et al., 2018b).

### 4.1 Osteogenic effects of calcium

**FIGURE 3:**
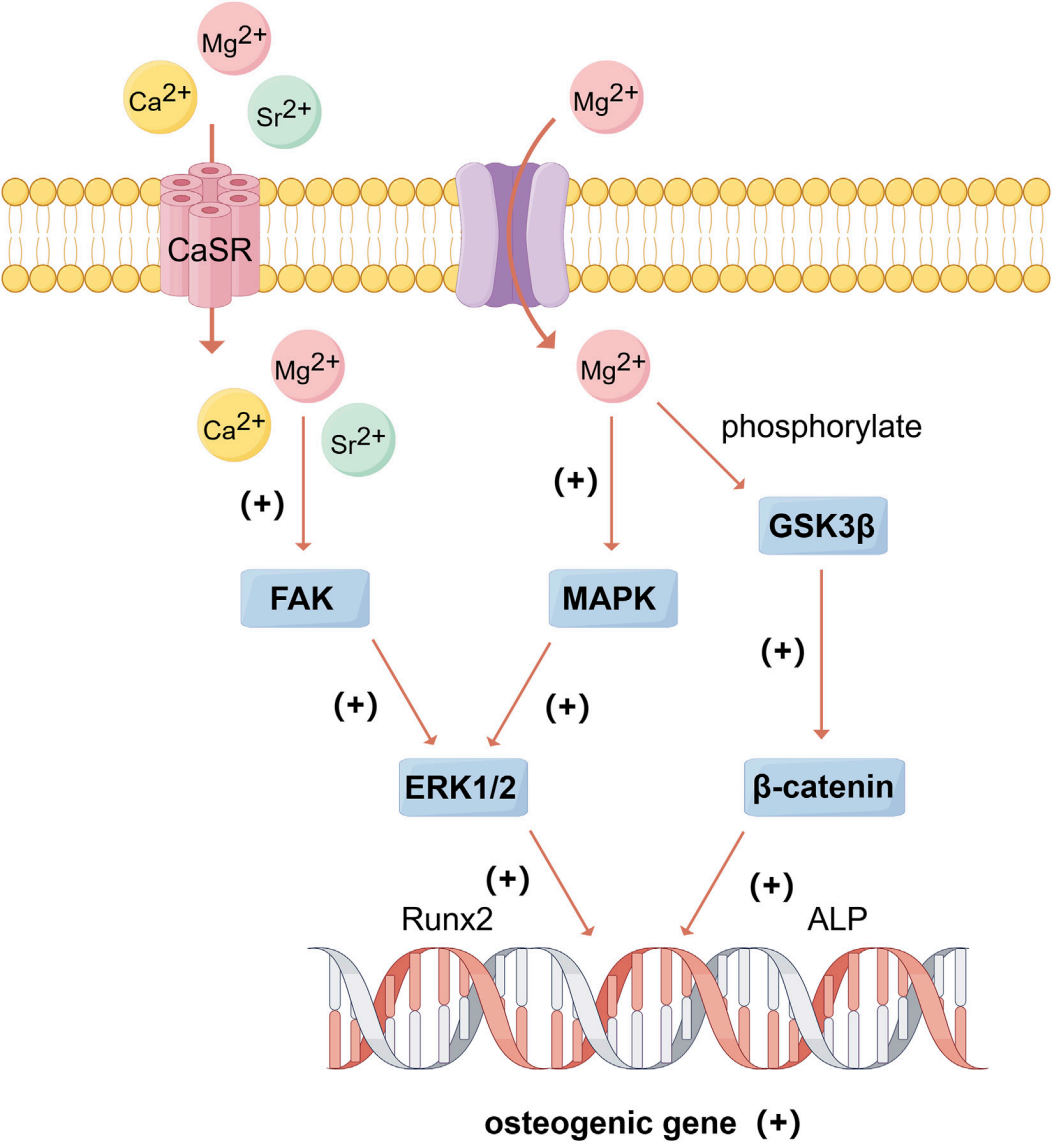
The beneficial effects of Mg, Ca, and Sr on bone formation. (By Figdraw). Mg^2+^ and Sr^2+^, as congeners of Ca^2+^, can bind to CaSR and activate FAK/ERK signaling pathway, and Mg^2+^ can trans-membrane transport into the cell and active MAPK/ERK and β-catenin signaling pathways, thus promoting the expression of osteogenic genes. González-Vázquez et al. (2014), Wan B. et al. (2020), Qi et al. (2021), Nie et al. (2022), Weng et al. (2023).

Ca significantly promotes bone formation. The osteogenic effect is primarily achieved by regulating several intracellular signaling pathways. Calcium ions (Ca^2+^) can bind to extracellular calcium-sensing receptors (CaSR), leading to the activation of tyrosine kinase, phospholipase C, and adenylate cyclase, which in turn triggers the phosphorylation of ERK 1/2, activates c-fos gene and mediates the expression of BMP-2 (Weng et al., 2023). This activation further stimulates the MAPK signaling pathway, promoting the mineralization of the extracellular matrix and the expression of osteogenic-related genes (González-Vázquez et al., 2014) (Figure 3). Additionally, Ca^2+^ can regulate the TGF-β/Smad and Wnt/β-catenin signaling pathways to enhance osteogenesis (Li S. et al., 2022; Liu Y. et al., 2023). The classical Wnt signaling pathway plays an important role in osteoblast differentiation. Wnt can bind to receptors and promote intracellular accumulation of β-catenin, which enters the nucleus and interacts with the transcription factor T cell factor/lymphatic enhancer (TCF/LEF) to activate the transcription of target genes, thereby inducing the expression of downstream osteogenic transcription factors such as Runx2 and Osterix (Wang Y. et al., 2019). Ca^2+^ can upregulate Wnt3a and Wnt co-receptor LRP5 (confirmed to be involved in osteogenic differentiation), thereby activate Wnt/β-catenin signaling pathways to promote osteogenesis (Wang Y. et al., 2019). Furthermore, studies have found that adding other metals with osteogenic properties, such as Zn and Sr, to Ca-containing materials can synergistically promote bone formation (Sutthavas et al., 2022).

### 4.2 Common preparation methods of biomaterials loaded with calcium

As a primary component of natural bone tissue, Ca plays a crucial intracellular role. Currently, various calcium phosphate-based biomaterials are widely used in orthopedic implants. Several studies have aimed to improve the osteogenic and antimicrobial activities of these materials using methods such as digital light processing (DLP), ion implantation, plasma surface modification, and photocrosslinking. Researchers have incorporated various metals such as Zn, Mg, Sr, Ag, and Co, either individually or in combination, into β-tricalcium phosphate (β-TCP) to enhance its stability, corrosion resistance, and to achieve stronger osteogenic and antimicrobial responses (Wan B. et al., 2020; Hurle et al., 2021; Li J. et al., 2021; Qi et al., 2022; Qian et al., 2022). Through the introduction of Ca^2+^ into the surface of Ti alloy implants by ion implantation, Huang et al. found that the formation of CaTiO_3_ nanoparticles on the surface of the material significantly improved the wettability, and the Ca^2+^ in the coating showed a continuous release trend in 14 days, which enhanced the adhesion, spreading, proliferation, and expression of osteogenic-related genes in MC3T3-E1 cells (Huang et al., 2022). Additionally, Ca can be synthesized through plasma surface modification methods or by combining it with other bioactive materials such as chitosan, gelatin, and hydroxyapatite (HA). These methods result in implant materials with improved mechanical properties, biocompatibility and osteoinductive properties (Choy et al., 2021; Zhang et al., 2022a; Zhao Z. et al., 2022). Tanzer et al. evaluated femoral remodeling of patients using titanium alloy femoral hip prostheses with or without HA-TCP coating for 2 years after total hip replacement, and dual-energy X-ray absorptiometry showed that bone mineral density (BMD) was significantly higher in the coated group, indicating that HA-TCP coating can significantly reduce bone loss in femur (Tanzer et al., 2001).

## 5 Magnesium

Mg is an essential electrolyte for living organisms and is the fourth most abundant mineral in the human body. Humans need regular intake of Mg to prevent Mg deficiency, and low levels have been linked to many chronic and inflammatory diseases, such as Alzheimer’s disease, asthma, attention deficit hyperactivity disorder, insulin resistance, type 2 diabetes, cardiovascular disease, and osteoporosis (Gröber et al., 2015). Mg is found mainly in cells, essential for maintaining normal vital activities and metabolism, it plays a significant role in neurotransmission, protein synthesis, the transport of potassium and calcium ions, and the phosphorylation of ATP and is indispensable for numerous biochemical reactions in the body (Glenske et al., 2018).

### 5.1 Osteogenic and angiogenic effects of magnesium

Mg, as a congener of Ca, shares several physicochemical properties and bone-contributing mechanisms with Ca. A deficiency of magnesium ions (Mg^2+^) in the blood, known as hypomagnesemia, may be a trigger for osteoporosis by the following pathways: (1) alter the structure of apatite crystals and affect bone cells; (2) reduce levels of parathyroid hormone (PTH) and lead to deficiency of vitamin D; (3) increase inflammatory cytokines and stimulate bone osteoporosis; (4) promote endothelial dysfunction. Therefore, proper magnesium concentration is essential for bone metabolism (Weng et al., 2023). For instance, Mg^2+^ regulate the expression of integrins and activates the Wnt/β-catenin signaling pathway (Figure 3). Glycogen synthase kinase-3β (GSK-3β) is a signaling protein and transcription factor that monitors cell growth, and β-catenin is a positive regulator of osteoblasts that promotes osteogenic differentiation and bone formation (Nie et al., 2022). Mg^2+^ has been found to promote new bone formation by inducing the phosphorylation of GSK-3β, which prevents the ubiquitination and degradation of β-catenin. This results in the accumulation of β-catenin in the cytoplasm and its subsequent translocation to the nucleus, where it upregulates Runx2 and OPG transcription, thereby activating the Wnt/β-catenin pathway (Qi et al., 2021). Studies have also shown that Mg^2+^ enhances the expression of osteogenic-related genes by up-regulating integrin expression in osteoblasts and activating focal adhesion kinase (FAK) and ERK signaling pathways, it can also promote the proliferation of MC3T3 cells by increasing ERK phosphorylation and enhancing c-fos levels (Nie et al., 2022).

Additionally, Mg^2+^ can upregulate the expression of angiogenesis-related genes. VHL protein can destroy the transactivation function of HIF through ubiquitin ligase, and the reducing expression of VHL can promote the function of HIF and angiogenesis (Kaelin, 2005). Mg^2+^ increases the expression of VEGF and HIF-1α and blocks VHL protein through modulating VHL/HIF-1α/VEGF signaling pathways, thereby enhance angiogenesis (Weng et al., 2023). Recent *in vitro* experiments have demonstrated that Mg^2+^ promotes the proliferation of human umbilical vein endothelial cells (HUVECs) and the expression of VEGF and endothelial nitric oxide synthase (eNOS), which are crucial for maintaining the survival of vascular endothelial cells (Gu et al., 2019; Zhang et al., 2021b). Li et al. prepared Mg-doped porous TiO_2_ coatings on Ti *via* MAO. Their study showed that coating-Ti upregulated the expression of the expression of growth factor genes (BMP-2 and VEGF), indicating that the coatings may endow Ti with osteogenesis and angiogenesis (Li et al., 2018c).

### 5.2 Common preparation methods of biomaterials loaded with magnesium

Mg and its alloys have been extensively used in bone tissue engineering due to their biodegradability and osteogenic effects. Common methods for preparing Mg-loaded biomaterials include HT, synthetic cross-linked coatings using phytic acid (PA), and melt and cast methods. Through the addition of Mg^2+^ to Ti implants to fabricate a PA-Mg coating by using PA as a cross-linking molecule, Liu et al. found that PA-Mg coating bound to the surface of Ti by covalent bond, improved the hydrophilicity of the surface of the material (possibly because the addition of Mg^2+^ increases the cation colonization on the surface) and promoted the adhesion of protein by electrostatic action. The PA-Mg-coated samples significantly promoted adhesion, proliferation, and osteogenic differentiation of BMMSCs with superior biocompatibility and osteoinduction compared to pure Ti samples (Liu et al., 2021). Chen et al. prepared nanoscale Mg/Cu metal-organic framework (MOF) coatings on pure Zn through HT. The addition of Mg-MOF made the material appeared super hydrophilic (possibly due to the presence of hydroxyl groups and porous spongy structures on the surface of the MOF), and the addition of Cu to MOF significantly improves the bonding strength between the coating and the substrate and the deformation resistance of the material. Benefit from the synergistic effect of local alkaline microenvironment generated by Zn^2+^, Mg^2+^ and Cu^2+^, the coatings significantly promoted osteoblast differentiation, enhanced the vascular formation of endothelial cells and showed excellent antibacterial activity against *S. aureus* and *E. coli* (Chen et al., 2024b).

Additionally, Canullo et al. used Mg-rich HA to fill alveolar cavities after tooth extraction to investigate its early angiogenesis and osteogenesis. Histology results from clinical trials revealed that the histological sections of the operative area were observed from dense connective tissue and capillaries covering to braided bone trabeculae, followed by extensive new bone formation and finally lamellar bone formation under the microscope, indicating that the material can effectively reduce alveolar ridge absorption, promote early angiogenesis and osteogenesis, and is a suitable material for preserving alveolar ridge (Canullo et al., 2013). Lee et al. used a biodegradable Mg alloy (Mg-5wt%Ca-1wt%Zn) for the treatment of hand and wrist fractures to investigate its clinical efficacy, biosafety, and feasibility. One-year clinical follow-up results showed that slow and controlled degradation of the implant occurred at the alloy-bone interface and it was completely replaced by the new bone within 1 year, which may avoid the need for secondary surgery and improve patient quality of life (Lee et al., 2016). However, clinical trials using Mg-coated materials to promote bone formation remains to be further studied.

## 6 Strontium

Although Sr is not an essential metal for the human body, it has the dual effect of promoting osteoblast activity and inhibiting osteoclast activity (Glenske et al., 2018; Wan B. et al., 2020). In organisms, Sr may be built into crystals of bioapatite and can form carbonates, citrates, and lactates. It can also bind to Ca transporters to replace Ca in certain physiological processes, such as muscle contraction, blood clotting, and the secretion of certain hormones (Kołodziejska et al., 2021). Strontium ranelate (SrRan) has been approved by the European Union for the treatment of postmenopausal osteoporosis since 2004, and Sr has gained increasing attention for its role in promoting osteogenesis (Liu et al., 2016).

### 6.1 Osteogenic and angiogenic effects of strontium

**FIGURE 4:**
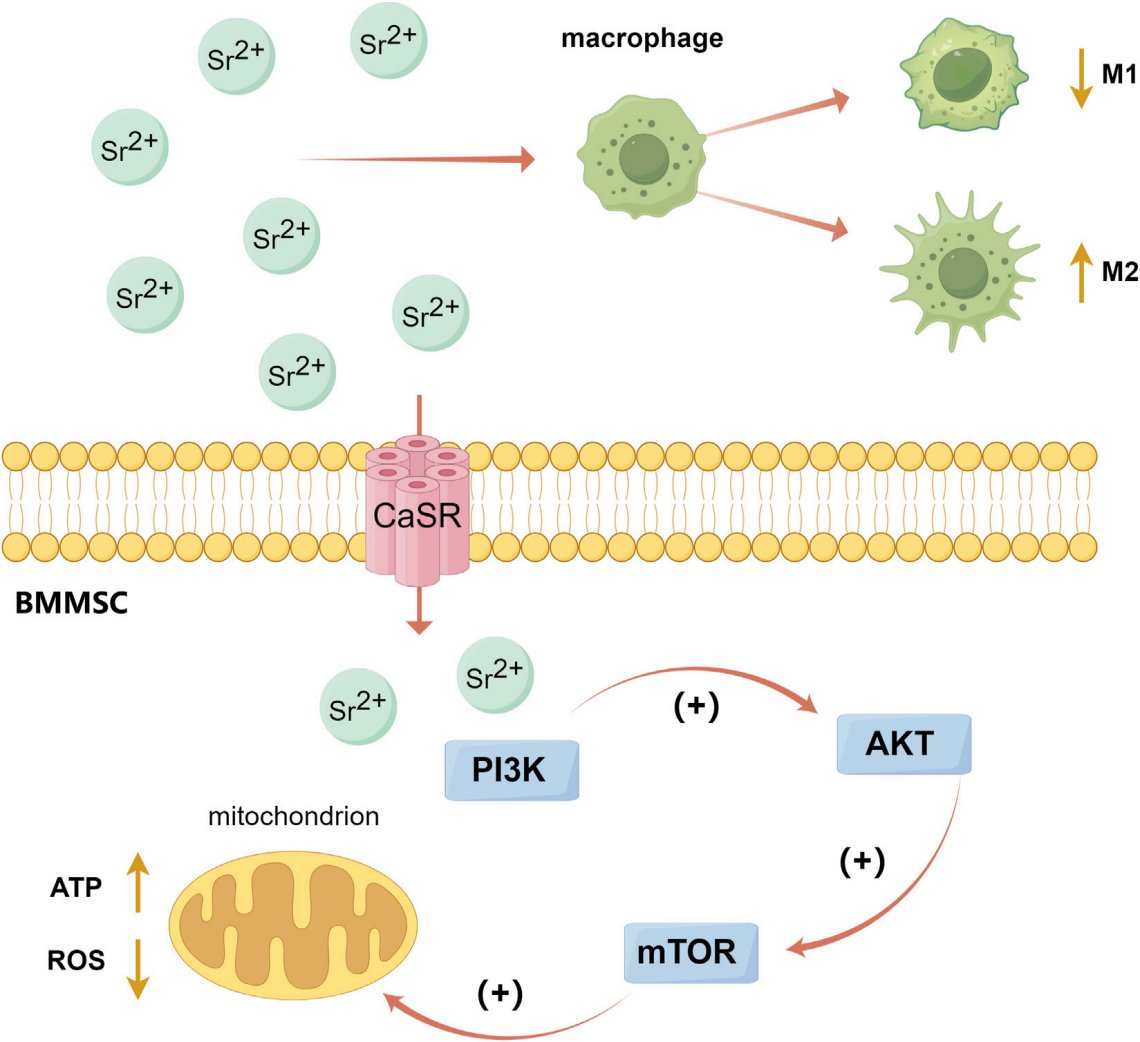
The PI3K/AKT/mTOR signaling pathway activated by Sr. (By Figdraw). Sr^2+^ can enhance the mitochondrial function of macrophages and activate the autophagy of BMMSCs by activating the PI3K/AKT/mTOR signaling pathway, thereby affect the polarization of macrophages and promote bone repair. Choi et al. (2014), Zhang et al. (2021a), Qiu et al. (2024).

Sr, an alkaline earth metal similar to Ca, can bind to calcium-sensing receptors (CaSR) and activate the intracellular Ca-driven MAPK signaling pathway (Wan B. et al., 2020) (Figure 3). Studies have shown that strontium ions (Sr^2+^) can also promote osteoblast differentiation by activating PI3K/AKT and Wnt/β-catenin signaling pathways (Li Y. et al., 2021; Sun et al., 2021; Wang et al., 2022). Phosphatidylinositol-3 kinase (PI3K) can activate protein kinase B (AKT or PKB) by converting phosphoinositide 4,5-bisphosphate (PIP2) to phosphoinositide 3,4,5-triphosphate (PIP3), and the activating AKT further regulates several downstream targets and participate in cell proliferation and metabolism (Stiles, 2009). The PI3K/AKT signaling pathway plays a role in cell metabolism and the M2 polarization of macrophages. One of AKT’s downstream targets is mammalian target of rapamycin (mTOR), which is involved in protein synthesis and cellular energy metabolism (Covarrubias et al., 2015). Autophagy can reduce ROS produced by mitochondrial respiration and improve bone integration by restoring cell proliferation and osteogenic differentiation (Zhang et al., 2021a). Studies have shown that Sr^2+^ can enhance the mitochondrial function of macrophages and activate the autophagy in BMMSCs by activating the PI3K/AKT/mTOR signaling pathway, thereby affecting macrophages polarization and promoting bone repair (Choi et al., 2014; Zhang et al., 2021a; Qiu et al., 2024) (Figure 4). Additionally, Sr promotes early angiogenesis by regulating the conversion of macrophages from M1 to M2 phenotypes, thereby accelerates the secretion of angiogenic factors, decreases the expression of proinflammatory factors (including iNOS, TNF-α, IL-1β and IL-6) and stimulates chondrogenesis by activating the HIF-1α signaling pathway (Wang C. et al., 2019; Cai et al., 2021). Lu et al. prepared Sr-coated micro/nano-titanium (SLA-Sr), which significantly promoted the migration and tube-forming behavior of HUVECs, regulated macrophage phenotype conversion, and increased mRNA levels of ALP, Col-1, and OPN in hBMMSCs, thus promoting osseointegration (Lu et al., 2022).

### 6.2 Inhibitory effects of strontium on osteoclasts

Sr can inhibit osteoclast formation and differentiation. Sr^2+^ significantly inhibits the expression of NFATc1 and downregulates the expression of osteoclast-related genes such as c-fos, TRAP, MMP-9, Cathepsin K, and TRAF6, thereby inhibiting osteoclast formation (Zeng et al., 2020). Carbonic anhydrase II (CA2), a key enzyme in bone resorption, and vitronectin receptor (VTNR), involved in cell capsule fold formation, can also be downregulated by Sr leading to the reduce of osteoclast differentiation and activity (Borciani et al., 2022). Sr can upregulate the expression of osteoprotegerin (OPG, which blocks the interaction between RANKL and RANK) and reduce the expression of RANKL, thus preventing excessive bone resorption (Bose et al., 2013; Lee et al., 2022) (Figure 1). Geng et al. prepared Sr-substituted apatite coating on pure Ti, and the results showed that TRAP activity and expression of NFATc1 and Cathepsin K in bone marrow monocytes (BMMs) on Sr-contained coatings were significantly decreased, indicating that Sr can effectively inhibit the activity of osteoclasts (Geng et al., 2021).

### 6.3 Common preparation methods of biomaterials loaded with strontium

Sr is primarily used to modify calcium phosphate ceramics (CaPs) or in combination with other metals to enhance osteogenesis. The modification of CaPs with divalent metal ions like Sr^2+^ can increase the material’s mechanical strength and biological activity. Study has demonstrated that Sr-substituted calcium phosphate ceramics (Sr-CaPs) positively affect new bone formation and accelerate bone healing (Wan B. et al., 2020). However, Sr lacks antibacterial properties, often necessitating its combination with Ag to achieve antimicrobial effects. Common preparation methods for Sr-loaded biomaterials include ionic substitution, the gel-sol process and MAO. Through the addition of Sr-Ag doped porous TiO_2_ coatings on Ti alloy by using MAO, Wang et al. found that the coatings showed well separated and uniformly distributed crater-like micropores. Sr presented on the coating in the form of SrTiO_3_, and Sr^2+^ exhibited explosive release in the first 7 days, then rapid release till day 30, and steady slow release in 120 days. The coatings showed excellent *in vitro* and *in vivo* antibacterial and osteogenic activity (Wang et al., 2023). Zhang et al. prepared Sr and Ag in porous TiO_2_ coatings on pure Ti *via* MAO which had a uniform distribution of pitted porous microstructure, imparting good osteogenic and antibacterial activities to the coatings without altering their microscopic morphology and physicochemical properties (Zhang Y.-Y. et al., 2021).

In terms of clinical application, strontium ranelate (SrRan) has been widely used in the treatment of osteoporosis in postmenopausal women, and clinical trial results showed that it can prevent cartilage loss, reduce bone marrow lesions and effectively reduce the risk of fracture (Seeman et al., 2010; Pelletier et al., 2015). Cheung et al. used a novel Sr-containing HA bioactive bone cement to replace the traditional polymethyl methacrylate (PMMA) bone cement, and the results showed that it exhibited better mechanical properties and biocompatibility *in vivo*, can promote the formation of osteoid layer and the inward growth of new bone *in vitro*, and it performed as effective as PMMA in pain relief in clinical trials. (Cheung et al., 2005). However, clinical trials using Sr-coated materials to promote bone formation remains to be further studied.

## 7 Silver

Ag is a non-essential element of the human body and is typically undetectable in the body under normal circumstances (Wu et al., 2023). Ag has now been widely used in biomedical fields such as drug delivery, medical imaging, dental antibacterial materials and wound dressings (Yin et al., 2020). The use of Ag in medical and commercial products has increased human exposure to Ag. Prolonged exposure to Ag (ions or nanoparticles) can lead to psoriasis, and inhaling Ag-containing aerosols or other particles can cause Ag to deposit in the lungs and/or be absorbed into the bloodstream (Betts et al., 2021).

### 7.1 Osteogenic effects of silver

The effect of silver ion (Ag^+^) on cellular activities is dose-dependent, and higher concentration of Ag^+^ will produce obvious cytotoxicity, so controlled release of Ag^+^ is crucial for achieving better cytocompatibility (Xiong et al., 2023). Nanoparticles (NPs), typically refer to particles with a size of 1–100 nm, may have different physical and chemical properties due to their high surface-to-volume ratio compared to bulk materials (Li et al., 2020). Compared with soluble Ag^+^ and bulk Ag, AgNPs have the advantage of controlled ions release, so that the Ag^+^ released from the material can be maintained at a level that does not produce cytotoxicity (Sun et al., 2023; Xiong et al., 2023).

AgNPs are thought to activate the TGF-β/BMP signaling pathway, which induce the chondrogenesis and osteogenic differentiation of MSCs, and induce the expression of HIF-1α to protect MSCs from hypoxia-induced cell death and promote fiber formation and end-junction of fractured bones (Zhang et al., 2015). Studies using microRNA (miRNA) and whole transcriptome technology have found that AgNPs can enhance the expression of Runx2 and Smad5 (both of which are essential osteogenic transcription factors and can activate bone-specific genes synergistically) in MC3T3-E1 cells to promote osteogenic differentiation (Mahmood et al., 2011; Qing et al., 2018). AgNPs are also able to induce cellular autophagy, which positively mediates osteogenesis of stem cells and osteoblasts. It has been shown that AgNPs can significantly increase the expression of LC3 and ubiquitin-binding protein p62 (both are autophagy related markers) which activated autophagy, and upregulate the expression of ALP, Col-1, OPN and OCN in MSCs, thereby leading to bone regeneration (He W. et al., 2020). Additionally, AgNPs promote the osteogenic differentiation of human periodontal ligament fibroblasts (HPDLFs) by activating the RhoA–TAZ signaling pathway. Activation of Ras homolog gene family member A (RhoA) stimulates the production of synthetic osteogenic growth peptides, and tafazzin (TAZ, a transcriptional coactivator associated with cell self-renewal, proliferation, and apoptosis) plays an important role in osteogenic differentiation (Xu et al., 2019). AgNPs can activate RhoA, induce downstream effector TAZ, and upregulate the expression of ALP, Runx2 and Col-1, thereby promoting the osteogenic differentiation of HPDLFs (Xu et al., 2019). Gao et al. used plasma immersion ion implantation (PIII) and MAO to prepare Ag-doped TiO_2_ nanotubes on the surface of Ti alloy implants, and the addition of Ag significantly promoted the mineralization of ECM and the production of ALP (Gao et al., 2023).

### 7.2 Antibacterial effects of silver

**FIGURE 5:**
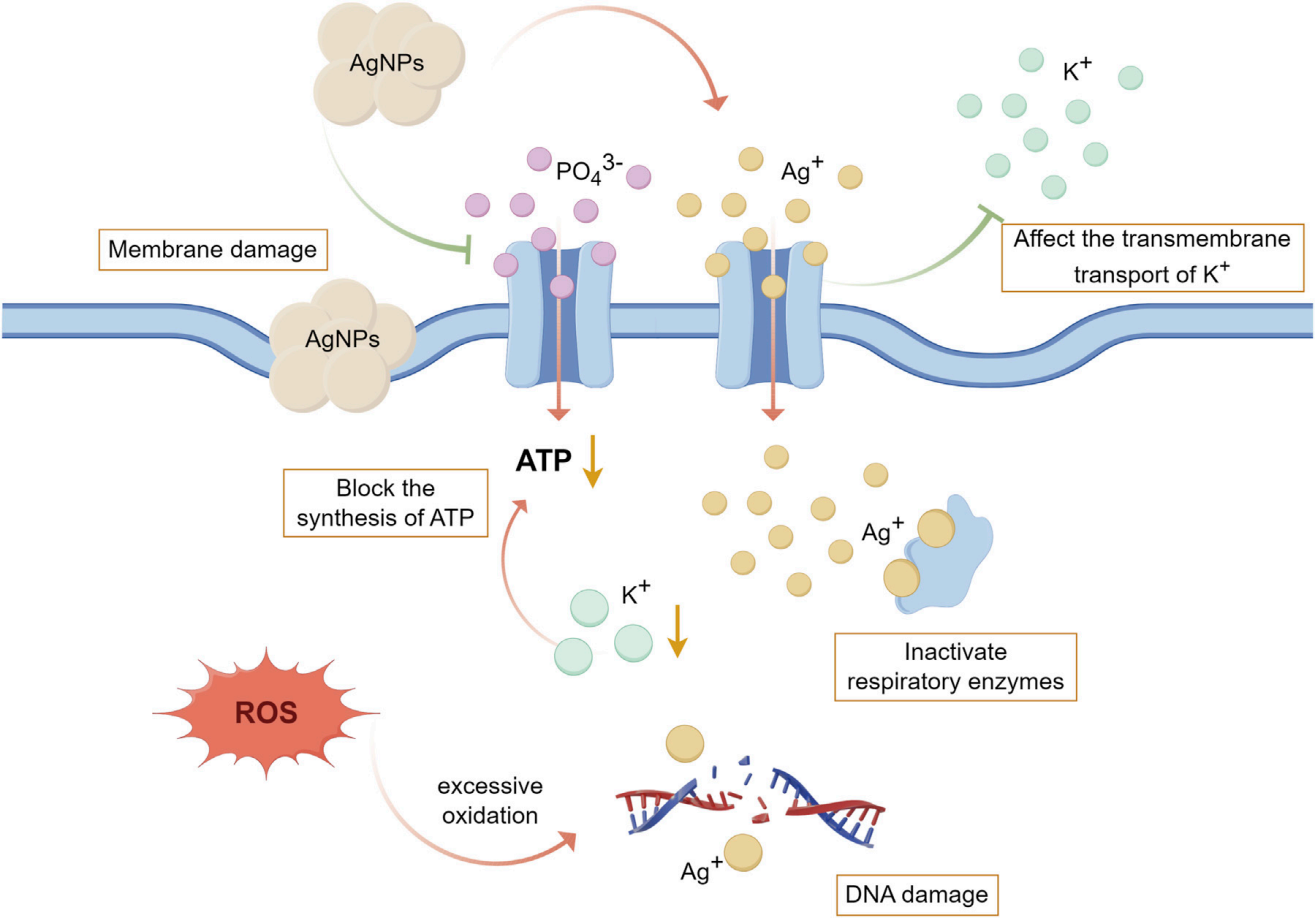
The antibacterial mechanism of AgNPs and Ag^+^. (By Figdraw). Firstly, AgNPs can destroy the structure of bacterial cell membrane, affect transmembrane transport, interfere with the uptake of PO_4_^3−^, destroy the respiratory chain, and hinder the production of ATP. Secondly, the Ag^+^ released from AgNPs can bind to the sulfhydryl group on the protein to inactivate the respiratory enzyme, affect the transmembrane transport of K^+^ thereby blocking the synthesis of ATP, and also affect DNA replication leading to the prevention of bacterial proliferation. Thirdly, AgNPs and Ag^+^ can induce the production of ROS and free radicals, leading to excessive oxidation of proteins, lipids and DNA. Durán et al. (2016), Tang and Zheng (2018), Yin et al. (2020), Li and Xu (2024).

Ag, as a broad-spectrum antibacterial agent, has remarkable antibacterial effect on both Gram-positive and Gram-negative bacteria, and the antibacterial effect of AgNPs is mainly achieved by the contact-killing mechanism and the release-killing mechanism (Nazarov et al., 2022). Firstly, in terms of the contact-killing mechanism, AgNPs can anchor and aggregate on the surface of bacteria, which causes the disruption of bacterial cell membrane and change the membrane structure, resulting in the breakdown of organelles and even cell lysis; AgNPs can bind to membrane proteins, increase membrane permeability and affect transmembrane transport; AgNPs, attached to membrane proteins, can also interfere with the uptake and release of phosphate ions and damage the respiratory chain, impeding the triphosphate (ATP) production of bacteria (Tang and Zheng, 2018; Yin et al., 2020). Secondly, the antibacterial effect of AgNPs is related to the release of Ag^+^, which can bind to sulfhydryl groups (involved in energy conversion during cellular respiration) on proteins to inactivate respiratory enzymes; and Ag^+^ can affect the transmembrane transport of potassium ions (K^+^) and block the synthesis of adenosine ATP; and Ag^+^ can also interact with sulfur and phosphorus in DNA to affect DNA replication and thus hinder the proliferation of bacterial (Durán et al., 2016; Tang and Zheng, 2018). Thirdly, AgNPs and Ag^+^ can induce the production of ROS and free radicals, excessive release of which will cause direct damage to mitochondrial membrane and lead to excessive oxidation of proteins, lipids and DNA, inhibit bacterial respiration and proliferation and eventually lead to bacterial death (Durán et al., 2016; Tang and Zheng, 2018; Li and Xu, 2024). (Figure 5) Sun et al. prepared double-layer TiO_2_ nanotube coatings loaded with AgNPs on the surface of Ti, which significantly inhibited the adhesion and proliferation of *P. gingivalis in vitro*, and also showed less bacteria signed by Giemsa staining in the rat model infected with *S. aureus* indicating its excellent antibacterial effect (Sun et al., 2023).

### 7.3 Common preparation methods of biomaterials loaded with silver

Benefiting from its strong inhibitory effect on a variety of bacteria, including antibiotic-resistant strains, Ag has been widely used in surface modification of implants to improve the antibacterial ability of materials (Qin et al., 2015). Through the addition of Ag-rich titanium nitride (TiN/Ag) nano-multilayer on Ti alloy, Wan et al. found that the hydrophilicity of the material has increased, and Ag^+^ in the nano-multilayer showed a stable and continuous release trend. Additionally, the Ti alloy coating showed a long-term stable inhibitory effect on *Staphylococcus epidermidis* and upregulated the ALP activity, ECM mineralization, and the expression of osteogenic-related genes of rBMMSCs without cytotoxicity (Wan R. et al., 2020). Zhang et al. utilized a flow-casting technique to prepare a bioceramic coating composed of 45S5 bioactive glass (BG) and core-shell Ag with mesoporous silica nanoparticles (Ag@MSN) on the surface of porous Ti (PT-BAg). Ag@MSN had a relatively low melting point and high activity, which increases the density of BG particles, significantly improved the adhesion strength of the coatings, and thus reduced the degradation rate of BG. Additionally, the dense PT-BAg coating could downregulate the release rate of Ag^+^, helping to improve the biocompatibility. *In vitro* experiments showed that PT-BAg can significantly inhibit *S*. *aureus*, and promote the proliferation and differentiation of MC3T3-E1 cells (Zhang et al., 2023). Additionally, AgNPs has been used in dental clinical applications for root canal irrigations, antibacterial coatings of gutta-percha, orthodontic ligatures, acrylic resins for removable partial dentures (Yin et al., 2020).

## 8 Cerium

Ce is a rare metal (lanthanide) occurring in the human body and is involved in stem cell differentiation and tissue regeneration (Liu X.-L. et al., 2023). Studies have shown that the depositions of Ce in the human bones are relatively high, indicating that bones may be the main site of cerium accumulation (Liu J. et al., 2024). Known for its pharmacological properties, Ce has been used as antiemetic, bacteriostatic and antitumor agent (Zambon et al., 2021). Cerium nitrate has attracted the most attention in the treatment of deep burns and is currently used in combination with silver sulfadiazine for wound treatment called Flammacerium (Barker et al., 2022a).

### 8.1 Osteogenic effects of cerium

**FIGURE 6:**
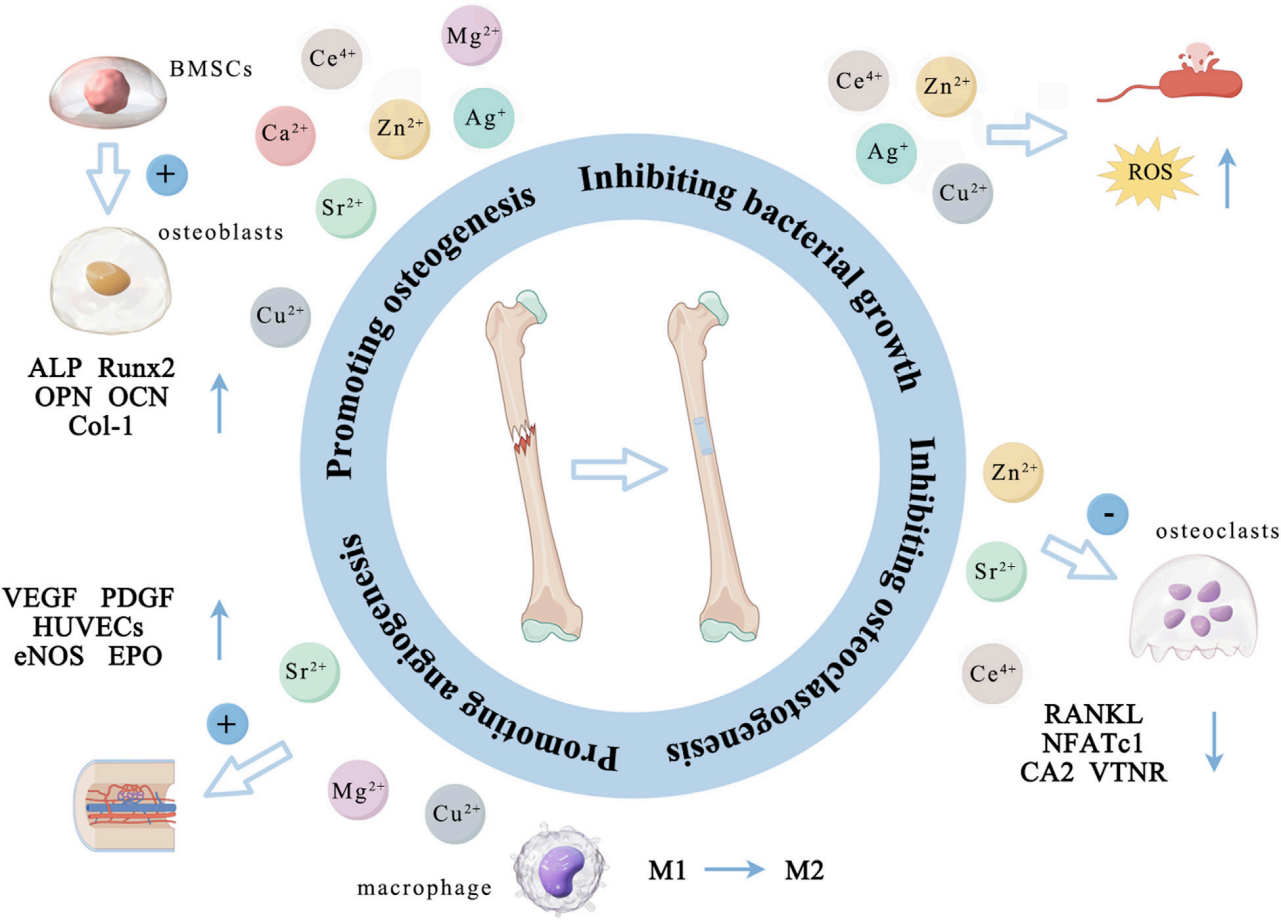
The main ways in which different metal ions promote osteogenesis. (By Figdraw). Zn^2+^, Cu^2+^, Ca^2+^, Mg^2+^, Sr^2+^, Ag^+^, and Ce^4+^ primarily promote the proliferation and differentiation of osteoblasts by activating several osteogenesis-related markers (ALP, OPN, OCN, Runx2 and Col-1). Zn^2+^, Sr^2+^, and Ce^4+^ indirectly support bone formation by down-regulating the expression of RANKL, NFATc1, CA2 and VTNR thus inhibiting osteoclast activity. Cu^2+^, Mg^2+^, and Sr^2+^ can promote angiogenesis by promoting the expression of VEGF, PGDF, eNOS and EPO and the cellular activity of HUVECs. Additionally, Zn^2+^, Cu^2+^, Ag^+^, and Ce^4+^ can inhibit bacterial activity by altering the bacterial microenvironment, interfering with bacterial cell metabolism, disrupting cell membranes, and inducing the production of ROS.

Ce exhibit catalytic activity in the form of elemental and solid oxides due to the low redox potential between Ce^3+^ and Ce^4+^, facilitating easy conversion between each other, and Ce exists in a mixed valence state between above two states in cerium oxide (CeO) (Wei et al., 2021a). CeO, as well as its NPs, have been extensively studied for their redox catalytic activity, including scavenging free radicals, which can reduce inflammatory responses, enhance osteoblast functionalization and promote new bone formation (Bao et al., 2024) (Figure 6). ROS, reactive oxygen species, are normal by-products of cellular oxidative metabolism and are functional in controlling cell and tissue homeostasis. However, oxidative stress caused by ROS excess can damage cellular structure and DNA and then disrupt bone regeneration and repair (Wei et al., 2021b). Studies have shown that CeO nanoparticles (CeO NPs) can mimic the activity of superoxide dismutase (Ce^3+^, Ce_2_O_3_) and catalase (Ce^4+^, CeO_2_) in catalytic reactions with superoxide and hydrogen peroxide, and can eliminate hydroxyl, nitric oxide and peroxynitrite radicals by simulating the properties of peroxidase, oxidase and phosphatase, thus inhibiting oxidative stress and inflammation and promoting the repair of defective tissue (Wei et al., 2021b; Zambon et al., 2021).

Additionally, Ce plays a role in promoting bone by activating several signaling pathways related to bone formation. Ce can bind to bone morphogenetic protein receptor (BMPR) and regulate the phosphorylation of Smad1/5/8, activate the Smad dependent BMP signaling pathway, upregulate the expression of Runx2, and subsequently promote the expression of Col-1, BMP-2, ALP and OCN (Liu et al., 2013; You et al., 2017). Ce can also promote nuclear translocation of β-catenin protein by increasing the expression of Fam53B gene, activate Wnt/β-catenin pathway, and promote differentiation of precursor osteoblasts (Luo et al., 2023). Li et al. produced CeO combined with calcium silicate coatings on Ti-6Al-4V, and the results showed that the inclusion of CeO significantly improved osteoblasts viability, reduced apoptosis caused by hydrogen peroxide, and increased ALP activity under H_2_O_2_-induced oxidative stress (Li K. et al., 2016).

### 8.2 Inhibitory effects of cerium on osteoclasts

Ce can inhibit osteoclast activity and indirectly promote bone formation by inhibiting ROS production. Increased ROS stimulates the expression of RANKL and tumor necrosis factor-α (TNF-α) through the activation of ERK and NF-κB, while decreasing the expression of OPG and promoting osteoclast formation and absorption (Wei et al., 2021a). Ce^3+^/Ce^4+^ can also act as an anti-inflammatory factor in macrophage polarization, inhibiting its differentiation towards pro-inflammatory phenotypes and osteoclasts, thereby reducing bone resorption (Yuan et al., 2020). Liu et al. constructed a nacre-mimetic Ce-doped hydroxyapatite/chitosan (CeHA/CS) layered composite scaffold and assessed its potential to modulate the osteogenic process and osteoclast differentiation. The results of *in vitro* experiments showed that the scaffold upregulated the expression of osteogenic genes (Runx2, Col-1 and OCN) in BMMSCs, inhibited the differentiation of bone marrow monocytes (BMMs) into osteoclasts and downregulated its TRAP activity, as well as the *in vivo* experiments showed that the RANKL/OPG ratio was significantly reduced, which indicated that CeHA/CS scaffolds significantly accelerate new bone formation through osteogenic promotion and osteoclast inhibition (Liu X.-L. et al., 2023). However, the inhibition of osteoclast activity by Ce-coatings remains to be further studied.

### 8.3 Antibacterial effects of cerium

Ce has also shown a basic and effective role in preventing bacterial adhesion and proliferation that can occur on implant surfaces. CeO NPs have recently been shown to have antibacterial effects against most Gram-positive and Gram-negative bacteria, including *P*. *aeruginosa*, *E*. *coli*, *Bacillus subtilis*, and *S*. *aureus* (Zamani et al., 2021; Barker et al., 2022b). However, the specific antibacterial mechanism of CeO NPs is still not completely clear. It is widely believed that CeO NPs can directly bind to bacterial and destroys its membrane, further penetrate into the cytoplasm and interfere with endogenous respiration and interact with phosphate compounds and proteins (Gavinho et al., 2022). Taking into account the characteristics of reversible transformation and reoxidation reaction between Ce^3+^/Ce^4+^, some studies considered that CeO NPs acts as a pro-oxidant (Ce^4+^) under low pH conditions, which is characteristic of the environment of bacterial infection or tumor tissue, and therefore Ce^4+^ is able to produce ROS and show antibacterial activity (Kurtuldu et al., 2021; Barker et al., 2022b; Chatzimentor et al., 2023). Li et al. produced nano-CeO coating on Ti surface to study its antibacterial properties, and the results showed that the coating had significant inhibitory effects on *Streptococcus haematococcus*, *P. gingivalis* and *Fusobacterium nucleatum* (Li et al., 2019).

### 8.4 Common preparation methods of biomaterials loaded with cerium

Considering the above characteristics of Ce’s osteogenic, antibacterial and catalytic activities, a large number of studies have incorporated Ce into biomaterials. Common preparation methods of Ce containing coatings include HT and plasma spraying. Through the addition of CeO-doped HA on Ti-6Al-4V using plasma spraying, Li et al. detected that the presence of CeO was in the form of Ce^3+^ and Ce^4+^ mixed valence states, and after immersing the material in simulated body fluids for 21 days they observed CeO significantly improved the apatite formation ability (Li et al., 2018a). Additionally, CeO coating also increased the cell viability of BMMSCs under H_2_O_2_ conditions and enhanced the formation of mineralized nodules and the expression of osteogenic genes (Li et al., 2018a). Bao et al. prepared nano-shaped CeO coatings on Ti alloy by HT, simulating the biological function of nano-enzymes used for free radical scavenging thus enhancing the antioxidant capacity of the material. Additionally, the coatings induced M2-polarized macrophages, which were beneficial to the regulation of bone immunology and vascularized bone integration, inhibited the inflammatory response and promoted the formation of neovascularization and new bone (Bao et al., 2024). Through the addition of CeO in the CS coating on Ti-6Al-4V using plasma spraying, Li et al. found that the chemical stability of CS coating was enhanced. Additionally, CeO played a better role than zirconium dioxide (ZrO_2_) coating (control group) in regulating the osteogenic activity of BMMSCs and the anti-inflammatory effect of RAW264.7 macrophages (Li et al., 2017).

## 9 Conclusion

The use of artificial bone materials with good osteogenic and antibacterial activities to repair large area bone defects is the focus of clinical research. At present, common metal ions such as Zn^2+^, Cu^2+^, Ca^2+^, Mg^2+^, Sr^2+^, Ag^+^, and Ce^4+^ are often loaded onto the surface of biological materials using various preparation methods due to their beneficial biological properties. The commonly used metal ion surface modification methods mainly include hydrothermal method, micro-arc oxidation, electrochemical deposition, ion implantation, plasma spraying and so on (Wang Z. et al., 2021). The raw material of hydrothermal method is cheap and easily accessible, and the obtained coating is of high purity and uniform distribution, but it has the disadvantages of long reaction time and high equipment requirements. Micro-arc oxidation can form a porous structure on the surface of the material that is conducive to bone bonding, but the coating is brittle and the reaction energy efficiency is high which limits its industrialization. Electrochemical deposition can be carried out at low temperatures and is suitable for heat-sensitive materials, but the coating is loose and the adhesion is poor. Ion implantation method has the advantages of controllable ion concentration and wide application range, but the preparation process requires vacuum environment and complex equipment. The coating obtained by plasma spraying has large thickness and good wear resistance, but it has high porosity and is prone to deformation under thermal stress, which requires follow-up treatment. According to different surface requirements, the corresponding surface modification methods can be selected to treat the substrate.

The above metal ions are known for promoting bone tissue growth and inhibiting bacterial activity (Tables 1 and 2). Zn^2+^, Cu^2+^, Ca^2+^, Mg^2+^, Sr^2+^, Ag^+^, and Ce^4+^ primarily promote the proliferation and differentiation of osteoblasts by activating osteogenesis-related markers (ALP, OPN, OCN, Runx2 and Col-1). Zn^2+^, Sr^2+^, and Ce^4+^ indirectly support bone formation by down-regulating the expression of RANKL, NFATc1, CA2 and VTNR thus inhibiting osteoclast activity. Cu^2+^, Mg^2+^, and Sr^2+^ can promote angiogenesis by promoting the expression of VEGF, PGDF, eNOS and EPO and the cellular activity of HUVECs (Figure 6; Table 3). Due to the lack of good osteogenic properties of artificial implant materials commonly used in clinical repair of bone defects (such as Ti and Ti alloy, PEEK, bone cement, *etc.*), the above metal ions can be added through surface modification to accelerate bone regeneration in the defect area after implantation. Additionally, bacterial infection after implantation of artificial bone material can lead to an inflammatory response around the implants, and even lead to implant loss and implantation surgery failure. Zn^2+^, Cu^2+^, Ag^+^, and Ce^4+^ can inhibit bacterial activity by altering the bacterial microenvironment, interfering with bacterial cell metabolism, disrupting cell membranes, and inducing the production of ROS (Figure 6; Table 3). As the “gold standard” of clinical repair of bone defect, autografts can significantly promote bone regeneration in the defect area without immune rejection. By introducing the above ions (Zn^2+^, Cu^2+^, Ca^2+^, Mg^2+^, Sr^2+^, Ag^+^, and Ce^4+^) into the artificial implant material, the bone binding ability of the material can be effectively improved while avoiding bacterial infection and immune response, thereby improving the success rate of implantation surgery, making it possible to become a potential alternative to autografts.

**TABLE 1:** Examples of osteogenic biomaterials produced by modification with metal ions.

Metal ions	Type of biomaterial	Experimental model	Effect on bone regeneration	References
Zn^2+^	Zn-doped TiO_2_ nanotube arrays, Zn-bound micro-Ti	*In vitro* model: MSCs, RAW 264.7	Improved the osseointegration of Ti implants, enhanced the proliferation and osteogenic differentiation of MSCs, inhibited the proliferation and activity of osteoclasts	Shen et al. (2014), Zhao et al. (2022a)
Zn^2+^, Ag^+^	Pure Zn, Zn-Ag alloys	*In vitro* model: bone marrow monocytes/macrophages (BMMs)	Reduced the differentiation of osteoclasts and bone resorption	Qu et al. (2021)
Zn^2+^	Zn-doped PEEK through plasma-induced surface modification	*In vitro* model: MC3T3-E1 cells	Promoted the adhesion, proliferation, and differentiation of MC3T3-E1 cells	Zhang et al. (2021c)
Zn^2+^, Sr^2+^	Sr-Zn co-doped mesoporous bioactive glass nanoparticles (Sr-Zn-MBGNs)	*In vitro* model: hMSCs	Enhanced mineralization, promoted bone regeneration and healing	Naruphontjirakul et al. (2024)
Cu^2+^	Cu-bearing titanium alloy with hierarchical structures	*In vivo* model: mouse osteomyelitis-model *In vitro* model: MC3T3-E1 cells	Increased the expression of osteogenic-related genes, promoted osteogenic differentiation and accelerated the formation of new bone around the implant *in vivo*	Liu et al. (2022)
Cu^2+^	GO/Cu nanocomposite coatings on chitosan/hyaluronic acid scaffolds	*In vitro* model: rBMMSCs *In vivo* model: rat subcutaneous bacterial infection model	Exhibited good cytocompatibility, promoting the osteogenic differentiation of rBMMSCs	Yang et al. (2022)
Cu^2+^, Mg^2+^	Cu, Mg co-doped TiO_2_ nanotube coatings on pure Ti	*In vitro* model: MC3T3-E1 cells and BMMSCs	Promoted the cell proliferation of MC3T3-E1 and the osteogenic differentiation of BMMSCs	Wang et al. (2021a)
Ca^2+^	Ca-coating Ti alloy (Ti-25Nb-3Mo-2Sn-3Zr)	*In vitro* model: MC3T3-E1 cells	Promoted the adhesion, spreading, proliferation, and expression of osteogenic-related genes from MC3T3-E1 cells	Huang et al. (2022)
Ca^2+^	Calcium nervonate nanoparticles	*In vivo* model: rat skull defect model *In vitro* model: MSCs	Promoted the osteogenic differentiation of MSCs, regulated both the local osteogenic and inflammatory microenvironments	Ma et al. (2023)
Mg^2+^	CSMP-MgO injectable hydrogels	*In vivo* model: rat critical-sized calvarial defected model *In vitro* model: MC3T3-E1 cells and HUVECs	Promoted osteoblast maturation of MC3T3-E1 cells. upregulated angiogenesis in HUVECs, promoted new bone formation *in vivo*	Chen et al. (2022)
Mg^2+^, Zn^2+^, Mn^2+^	Mg-3Zn alloy, and Mg-2Zn-1Mn alloy	*In vivo* model: rat femoral fracture model *In vitro* model: rBMMSCs	Promoted the expression of bone morphogenetic protein and fibroblast growth factor and showed osteogenesis-promoting effects *in vivo*	Li et al. (2022a)
Sr^2+^	Sr-encapsulated micro/nano-titanium	*In vivo* model: rat tibial implant model *In vitro* model: hBMMSCs, HUVECs and Raw264.7	Promoted the migration and tube-forming behavior of HUVECs, regulated the conversion of macrophages from M1 to M2 phenotypes, increased osteogenic mRNA levels and showed vascularized osseointegration *in vivo*	Lu et al. (2022)
Sr^2+^	Sr-silk fibroin co-assembly hydroxyapatite nanoparticles (Sr-SF-HA)	*In vivo* model: rat subcutaneous pocket model *In vitro* model: rBMMSCs and HUVECs	Exhibited good osteogenic induction ability, promoted angiogenesis and ectopic osteogenesis *in vivo*	Liu et al. (2024b)
Ag^+^	silver-rich Ti nitride nano-multilayer onTi-6Al-4V	*In vivo* model: SD rats *In vitro* model: rBMMSCs	Promoted the osteogenic differentiation of rBMMSCs, and exhibited good biosafety *in vivo*	Wan et al. (2020b)
Ce^3+^/Ce^4+^	nano-shaped CeO coating-Ti	*In vivo* model: rat femoral condyles model *In vitro* model: RAW264.7, HUVECs and MC3T3-E1	Induced M2-polarized macrophages, and inhibited the inflammatory response and promoted the formation of neovascularization and new bone *in vivo*	Bao et al. (2024)

**TABLE 2:** Examples of antimicrobial biomaterials produced by modification with metal ions.

Metal ions	Type of biomaterials	Antibacterial activity	Possible antibacterial mechanisms	Effect on eukaryotic cells	References
Zn^2+^	Zn-substituted β-TCP	*Enterococcus faecalis*, *E. coli*, and *Pseudomonas aeruginosa*	The antibacterial activity is expressed through contact killing.	Biocompatibility (studies on NCTC L929 fibroblast cells from murine subcutaneous connective tissue)	Fadeeva et al. (2021)
Cu^2+^	Cu-doped nano-scale thin film on the Ti-6Al-4V	Methicillin-resistant *S. aureus*	The antibacterial activity is expressed through contact killing.	Biocompatibility (studies on MC3T3-E1)	Shimabukuro et al. (2024)
Cu^2+^	Cu-doped TiO_2_ microporous coatings	*S. aureus and P. gingivalis*	Disrupt the bacterial cell membranes and result in protein denaturation and cell death.	Biocompatibility and osteogenic ability (studies on BMMSCs)	kang et al. (2022)
			Induce oxidative stress by the generation of ROS and inhibit the growth of bacterial.		
Zn^2+^, Sr^2+^	Sr-Zn-MBGNs	*E. coli* and *S. aureus*	Influence microbial metabolic processes, including alterations in osmolarity, the formation of ROS, and changes in pH levels.	Biocompatibility and osteogenic ability (studies on hMSCs)	Naruphontjirakul et al. (2024)
Ag^+^	double-layer TiO2 nanotube coatings loaded with AgNPs	*P. gingivalis* and *S. aureus*	The antibacterial activity is expressed through contact killing and the release of Ag^+^ and ROS.	Biocompatibility and osteogenic ability (studies on rBMMSCs)	Sun et al. (2023)
Ce^3+^/Ce^4+^	CeO coating pristine/nanotubular-structured Ti and Ti-6Al-4V	*S. aureus* and *P. aeruginosa*	Induce oxidative stress by the generation of ROS and inhibit the growth of bacterial.	Biocompatibility (studies on human fetal lung immortalized fibroblasts)	De Santis et al. (2023)

**TABLE 3:** Different metal ions and their outcomes. (+, promotes; −, inhibits; na, not known).

Metal ions	Osteogenic differentiation	Angiogenesis	Osteoclastogenesis	Antibacterial property
Zn^2+^	+	na	−	+
Cu^2+^	+	+	na	+
Ca^2+^	+	na	na	na
Mg^2+^	+	+	na	na
Sr^2+^	+	+	−	na
Ag^+^	+	na	na	+
Ce^4+^	+	na	−	+

However, the osteogenic and angiogenic mechanisms of individual metal ions are complex, and the concentration of metal ions released from the material is crucial for promoting bone formation. The interaction between different metal ions and various types of biomaterials is also a significant factor influencing the success of implantable materials. Therefore, in developing new materials, further research is needed to determine the optimal metal ion concentration, the best combination of different metal ions and the compatibility of different biomaterials with these ions. Additionally, extensive *in vivo* experiments are necessary to confirm the biosafety, osteogenic and antibacterial properties of these metal ions. Research on the application of metal ions surface modification in clinical practice is still relatively lacking, and it is hoped that this review can provide reference for clinical research and application of biomaterials with osteogenic and antibacterial activities.
